# Refractive changes after strabismus surgery in patients with intermittent exotropia

**DOI:** 10.1371/journal.pone.0280274

**Published:** 2023-01-12

**Authors:** Yeji Moon, Seong-Joon Kim

**Affiliations:** 1 Department of Ophthalmology, Asan Medical Center, University of Ulsan College of Medicine, Seoul, Republic of Korea; 2 Department of Ophthalmology, Seoul National University Hospital, Seoul, Republic of Korea; 3 Department of Ophthalmology, Seoul National University College of Medicine, Seoul, Republic of Korea; Cairo University Kasr Alainy Faculty of Medicine, EGYPT

## Abstract

**Objectives:**

To evaluate the long-term refractive changes after horizontal muscle surgery in patients with intermittent exotropia and investigate the correlation between changes in the postoperative refractive error and clinical factors.

**Methods:**

We retrospectively reviewed the clinical data of patients aged < 15 years who underwent unilateral strabismus surgery (lateral rectus recession and medial rectus resection [RR, n = 47], lateral rectus recession and medial rectus plication [RP, n = 81], or lateral rectus recession [LRc, n = 68]). Preoperative and postoperative refractive errors up to four years after surgery were recorded. A mixed model was applied to compare the refractive error between the operated and fellow eyes and identify the factors associated with postoperative refractive changes.

**Results:**

The mean age at surgery was 7.5±2.4years, and girls accounted for 56.1% of the study population. There was no significant difference in the change in the spherical equivalent of refractive error between both eyes throughout the postoperative period. In contrast, the operated eyes consistently and significantly showed higher cylindrical power in with-the-rule astigmatism by 0.25D than in fellow eyes. Age, sex, and preoperative refractive error were not correlated with changes in postoperative astigmatism. Meanwhile, the type of surgery showed a significant interaction with the astigmatism changes. RP had less effect on the changes in astigmatism than RR and LRc (*p* = 0.001 and *p* = 0.022, respectively).

**Conclusions:**

Horizontal muscle surgery has no long-term effect on the change in the spherical equivalent. However, mild with-the-rule astigmatism is induced and sustained after surgery, and the type of surgery affects the postoperative change of astigmatism.

## Introduction

Surgery is a major treatment for intermittent exotropia (IXT), a common form of childhood ocular misalignment [1, 2]. The surgical procedures for treating IXT are composed of lateral rectus muscle (LR) weakening, such as recession and medial rectus muscle (MR) strengthening, including resection and plication. A procedure on the horizontal extraocular muscle can correct exodeviation by altering the action of the extraocular muscle. However, it can also cause changes in the refractive error by reducing the tension of the extraocular muscle transmitted through the sclera to the cornea [3–6].

Several previous studies have described changes in spherical equivalent after horizontal muscle surgery. Most of them reported a postoperative myopic shift [6–10], although some showed a hyperopic shift or no significant change in spherical equivalent [11, 12]. As with the spherical equivalent, an increase in with-the-rule (WTR) astigmatism has been reported in the majority of studies [6–9, 13, 14]. However, previous studies focused on the immediate refractive changes within up to one year after surgery. Therefore, the long-term implications of strabismus surgery on refractive error remain unclear.

In addition, the magnitude of refractive changes and their clinical significance remains controversial. This is in part because refractive error can be affected by various biogenetic and environmental factors. Therefore, potential confounding factors should be controlled or eliminated to assess the true effect on refractive error. Several studies on refractive error in the patients with strabismus have been performed with a paired comparison between the two eyes to control for these confounding factors [14, 15].

This study aimed to evaluate refractive changes after unilateral horizontal muscle surgery in patients with IXT by comparing the operated and fellow eyes to deduce the long-term implications of strabismus surgery on refractive error. Furthermore, we investigated the correlation between changes in postoperative refractive error and clinical factors, such as surgical amount and surgical technique.

## Materials and methods

The study protocol was approved by the institutional review board of Seoul National University Hospital. All data were fully anonymized and the requirement for informed consent was waived owing to the retrospective study design. The study was conducted in accordance with the tenets of the Declaration of Helsinki.

### Study subjects

This was a retrospective review of the medical records of patients under 15 years of age who underwent unilateral strabismus surgery (lateral rectus recession and medial rectus resection (RR), lateral rectus recession and medial rectus plication (RP), or lateral rectus recession (LRc)) for IXT between January 2009 and December 2018 at Seoul National University Children’s Hospital in South Korea. The following exclusion criteria were applied: (1) best-corrected visual acuity worse than 0.3 logMAR (20/40) in at least one eye; (2) concurrent intraocular diseases such as congenital cataract, glaucoma, retinal disease, and optic atrophy or abnormal lid conditions such as ptosis; (3) previous history of strabismus surgery or lid surgery; (4) horizontal rectus muscle surgery with vertical transposition; (5) any combined vertical rectus or oblique muscle surgery; (6) a previous history of treatment for myopia control; and (7) a history of syndromic disorders known to be associated with myopia, such as Stickler syndrome, Marfan syndrome, and Knobloch syndrome.

### Preoperative ophthalmic examinations

We reviewed the patients’ medical records to obtain basic demographic data and ophthalmological examination results. We performed full preoperative ophthalmological examinations, including measurement of best-corrected visual acuity, cycloplegic refraction, deviation angle, stereoacuity, testing for fusional ability, slit-lamp examination, and fundus examination.

For measurement of preoperative refraction, cycloplegia was induced by three cycles of one drop of 1% cyclopentolate and one drop of 1% tropicamide, administered 5 min apart. After confirming the cycloplegic state 30 min after the last cycle of cycloplegic eye drops, manual retinoscopic refraction was performed. Measurement of refraction during the follow-up period after surgery was performed using non-cycloplegic refraction. Manual retinoscopic refraction was performed by our senior author (SJK). The cylindrical power in WTR astigmatism was recorded as minus (-) numbers and against-the-rule astigmatism as plus (+) numbers. Refractive error was measured with an accuracy of ±0.25D. Postoperatively, manifest refraction was assessed at every visit, and postoperative refractive data were obtained up to four years after surgery.

Extraocular motility testing was conducted, including the duction and version test, prism test, and alternating cover test. The angle of deviation was measured in five cardinal position (6 m) and near (0.33 m) as well as in both head tilt positions by a single experienced ophthalmologist (SJK). In addition, monocular occlusion test was performed at least once before surgery. The duration of the monocular occlusion was more than 30 minutes. Post-occlusion angle of deviation was measured before reestablishment of binocularity. Binocular function was tested using the Worth four-dot test and stereoacuity was measured using the Titmus stereo test (Stereo Optical, Chicago, Illinois, USA) when cooperation was adequate.

### Surgical procedures

All procedures were performed by a single surgeon (SJK) under general anaesthesia. To determine the preferred eye for fixation, a repeated cover-uncover test was performed for each patient. Surgery was performed on the non-dominant eye in patients who showed fixation preference. When a fixation preference was not observed, surgery was performed on one randomly selected eye.

The surgical dose was determined based on the surgeon’s experience, considering the largest angle of distance deviation (Table 1). A fornix conjunctival incision was made, and recession of the lateral rectus muscle was performed first, followed by a procedure on the MR during RR or RP.

**Table 1:** Surgical table in children with intermittent exotropia.

Deviation (PD)	RR group		RP group		LRc group
	LR recession (mm)	MR resection (mm)	LR recession (mm)	MR plication (mm)	LR recession (mm)
**15**					9.0
**20**	5.5	4.0	5.5	5.5	9.5
**25**	6.0	4.5	6.0	6.0	10.0
**30**	6.5	5.0	6.5	6.5	
**35**	7.5	5.5	7.5	7.5	
**40**	8.0	6.0	8.0	8.0	
**45**	8.5	6.5			
**50**	9.0	6.5			

RR, lateral rectus recession and medial rectus resection; RP, lateral rectus recession and medial rectus plication; LRc, lateral rectus recession; PD, prism dioptre; LR, lateral rectus; MR, medial rectus.

In cases of RR, the desired amount of resection was measured from muscle insertion, and locking bites were made using two sing-armed 6–0 polyglactin 910 sutures. The MR was disinserted and resected. The resected muscle was reattached to the sclera. To prevent muscle slippage during tightening, each needle was intrasclerally passed from the edge of the stump to the centre. Finally, the needles were brought through the centre between the two locking bites of the resected muscle and the suture was tied.

The plication procedures have been described in a previous study [16]. Briefly, locking bites using two single-armed 6–0 polyglactin 910 sutures were made away from the muscle insertion using the desired amount of plication. These sutures were then attached to the sclera immediately anterior to the edges of the original muscle insertion.

### Statistical analysis

Continuous data were summarised and presented as the mean ± standard deviation and range, while categorical data were presented as proportions and percentages. A paired t-test and analysis of variance were used to compare preoperative clinical characteristics. A mixed model was applied to evaluate the difference in longitudinal changes of refractive error between both eyes throughout the postoperative follow-up period and identify the factors associated with postoperative changes in refractive errors [17]. A mixed model is a statistical model containing fixed and random effects, so that it is useful for analysis of repeated measurements such as longitudinal data. It also allows to specify factorial interactions. All statistical analyses were performed using SAS version 9.4 (SAS Institute Inc., Cary, North Carolina, USA), and *p*-values of < 0.05 were considered significant.

## Results

The baseline demographic and clinical data are summarised in Table 2. The mean age at surgery was 7.5 ± 2.4 years, and 56.1% of the patients were female. The deviation angle of the RR group (30.9 ± 6.0 PD at distance, and 35.9 ± 6.3 PD at near) was significantly larger than the RP group (25.8 ± 4.8 PD at distance, and 32.8 ± 4.9 PD at near), which was larger than the LRc group (21.2 ± 3.5 PD at distance, and 22.9 ± 5.3 at near). Accordingly, there was a significant difference in the amount of surgeries between the three groups. The amount of LR recession in the LRc group was 9.4 ± 0.3 mm, which was the largest among the three groups, followed by the RR group (6.3 ± 1.1 mm) and finally the RP group (5.9 ± 0.5 mm). However, there was no significant difference in stereopsis or binocular function among the groups.

**Table 2:** Baseline demographic data and clinical characteristics of the study population.

Variables	Total	RR	RP	LRc	*p*-value
	(n = 196)	(n = 47)	(n = 81)	(n = 68)	
**Age at surgery (years)**	7.5 ± 2.4	7.2 ± 2.6	7.5 ± 2.3	7.7 ± 2.3	0.417
**Sex (male:female)**	86:110	24:23	34:47	28:40	0.530
**Operative eye (right:left)**	83:113	15:32	41:40	27:41	0.104
**Deviation angle (PD)**					
at distance*	25.4 ± 6.0	30.9 ± 6.0	25.8 ± 4.8	21.2 ± 3.5	**< 0.001**
at near*	30.1 ± 7.6	35.9 ± 6.3	32.8 ± 4.9	22.9 ± 5.3	**< 0.001**
**Stereopsis (Log arcsec)**	4.58 ± 1.12	4.84 ± 1.27	4.44 ± 0.98	4.58 ± 1.14	0.171
**Binocular function (F:S:D)**					
**at distance**	52:116:11	12:28:1	27:43:5	13:45:5	0.373
**at near**	109:49:21	29:8:4	42:24:9	38:17:8	0.699
**Amount of surgery (mm)**					
**LR recession^†^**	7.2 ± 1.7	6.3 ± 1.1	5.9 ± 0.5	9.4 ± 0.3	**< 0.001**
** MR resection/plication**	5.8 ± 0.8	5.1 ± 0.4	6.2 ± 0.6	n/a	**< 0.001**

Abbreviations: RR, unilateral lateral rectus recession-medial rectus resection; RP, unilateral lateral rectus recession-medial rectus plication; LRc, unilateral lateral rectus recession; PD, prism diopter; F, fusion; S, suppression; D, diplopia; LR, lateral rectus; MR, medial rectus.

*Post-hoc analysis reveals that RR > RP > LRc (all *p*-values < 0.0167).

^†^Post-hoc analysis revealed that LRc > RR > RP (all *p*-values < 0.0167).

The eyes that underwent surgery were more myopic than the fellow eyes in the preoperative period (-1.06 ± 2.09 vs -0.89 ± 1.90, p = 0.020). However, there was no significant difference in astigmatism between the eyes (Table 3).

**Table 3:** Preoperative refractive errors in both eyes.

Preoperative refractive error (D)	Total	RR	RP	LRc	*p*-value*
	(n = 197)	(n = 48)	(n = 81)	(n = 68)	
**Spherical equivalent**					
** Operated eye**	-1.06 ± 2.09	-1.36 ± 2.40	-0.74 ± 2.00	-1.23 ± 1.94	0.191
** Fellow eye**	-0.89 ± 1.90	-1.16 ± 2.25	-0.65 ± 1.77	-0.98 ± 1.78	0.302
*p*-value^†^	**0.020**	0.105	0.408	0.092	
**Cylinder**					
** Operated eye**	-0.78 ± 1.05	-0.76 ± 0.89	-0.75 ± 0.97	-0.83 ± 1.24	0.877
** Fellow eye**	-0.77 ± 1.00	-0.80 ± 0.83	-0.70 ± 0.92	-0.83 ± 1.19	0.707
*p*-value^‡^	0.757	0.495	0.268	0.947	

Abbreviations: RR, unilateral lateral rectus recession-medial rectus resection; RP, unilateral lateral rectus recession-medial rectus plication; LRc, unilateral lateral rectus recession; D, diopter.

*Analysis of variance among three (RR, RP and LRc) groups.

^†^Paired t-test between both eyes for the spherical equivalent.

^‡^Paired t-test between both eyes for the cylindrical power.

### Changes in the spherical equivalent of refractive error

**Fig 1:**
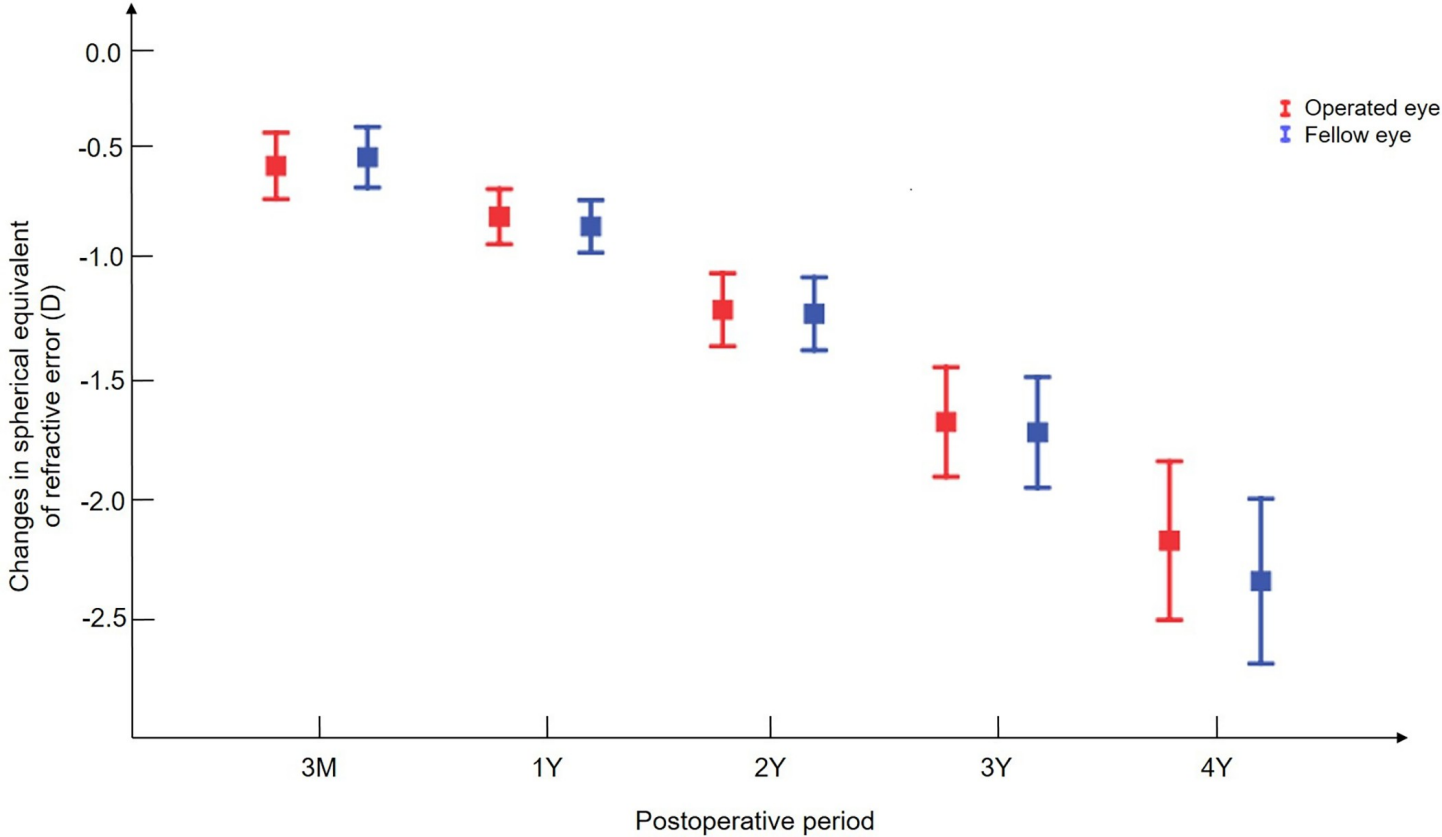
Changes in the spherical equivalent of the refractive error of both eyes over time. The course of the operated eyes is presented as blue lines and that of the fellow eyes as red lines. The error bar indicates the standard error of the mean.

In the mixed model analysis, there was no significant difference in the change in the spherical equivalent of refractive error (SER) between both eyes throughout the postoperative period from three months after surgery (**Fig 1**). SER error consistently decreased after strabismus surgery in both eyes, and the estimated rate of myopic progression was -0.49 D/year (95% CI, -0.53 - -0.45 D/year).

### Factors associated with changes in the SER error

Further mixed model analysis for testing the interaction showed that there were no factors associated with the change in SER after surgery. Both eyes showed no significant difference in changes in SER after strabismus surgery, regardless of age, sex, preoperative SER and cylindrical power, surgery type, and amount of surgery.

### Changes in astigmatism

**Fig 2:**
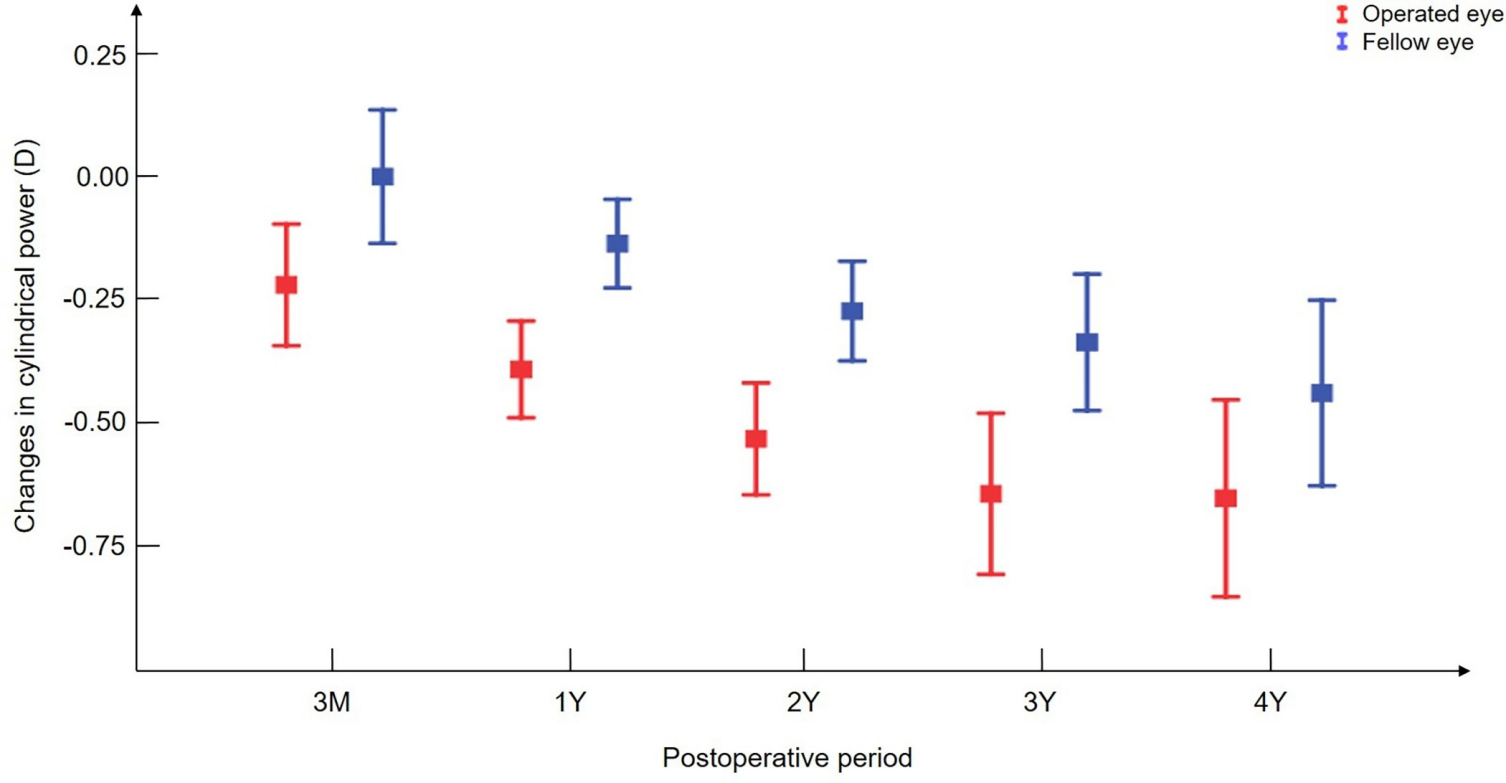
Changes in the cylindrical power of both eyes over time. The course of the operated eyes is presented as blue lines and that of the fellow eyes as red lines. The error bar indicates the standard error of the mean.

**Fig 2** presents the postoperative changes in astigmatism from three months after the surgery. Both eyes showed increased WTR astigmatism over time. The operated eyes consistently and significantly showed higher cylindrical power in WTR astigmatism than the fellow eyes. The difference in astigmatism between both eyes was 0.25 D (95% CI, 0.19–0.31 D), and the magnitude of difference did not significantly change with time (*p* = 0.984).

### Factors associated with changes in astigmatism

A linear mixed model analysis with interaction revealed that there was no significant effect on the difference in postoperative cylindrical power between both eyes when age, sex, preoperative SER, and cylindrical power changed, which means that these factors were not correlated with changes in postoperative astigmatism.

The type of surgery showed a significant interaction with the difference in postoperative astigmatism between eyes (Table 4). The eyes that underwent RR or LRc showed greater changes in astigmatism than those that underwent RP (RR vs RP, *p* = 0.001 and RP vs LRc, *p* = 0.022, respectively). Meanwhile, there was no significant difference in astigmatism changes after surgery in the fellow eyes among the groups. The operated eyes after RR showed greater changes toward WTR astigmatism by 0.41 D (95% CI, 0.30–0.52 D) compared to the fellow eyes (*p* < 0.001). The difference in astigmatism between both eyes was 0.10 D (95% CI, 0.01–0.19 D) in the RP group (*p* = 0.032) and 0.31 D (95% CI, 0.22–0.41D) in the LRc group (*p* < 0.001), respectively.

**Table 4:** Differences in postoperative astigmatism according to the type of strabismus surgery (A) in the total patients and (B) in the patients whose deviation angel was ≧ 20 PD and < 40 PD.

**(A)**						
	Least squares means (95% CI)			*p*-value		
	RR	RP	LRc	RR vs RP	RR vs LRc	RP vs LRc
**Operated eye**	-0.65 (-0.80 –-0.49)	-0.32 (-0.43 –-0.20)	-0.52 (-0.65 –-0.39)	**0.001**	0.210	**0.022**
**Fellow eye**	-0.24 (-0.39 –-0.09)	-0.22 (-0.34 –-0.10)	-0.21 (-0.33 –-0.08)	0.812	0.721	0.887
*p*-value	**< 0.001**	**0.032**	**< 0.001**			
**(B)**						
	**Least squares means (95% CI)**			***p*-value**		
	RR	RP				
**Operated eye**	-0.68 (-0.84 –-0.52)	-0.31 (-0.43 –-0.20)		**< 0.001**		
**Fellow eye**	-0.27 (-0.43 –-0.11)	-0.20 (-0.32 –-0.08)		0.491		
*p*-value	**< 0.001**	**0.016**				

Abbreviations: RR, unilateral lateral rectus recession-medial rectus resection; RP, unilateral lateral rectus recession-medial rectus plication; LRc, unilateral lateral rectus recession.

We additionally compared the postoperative astigmatism change between the RR and RP groups only in the patients whose deviation angle was ≧ 20 PD and < 40 PD. In this subgroup analysis, the amount of LR recession was not difference between both subgroups (6.0 ± 0.8 mm vs 5.9 ± 0.4 mm, *p* = 0.273), whereas the amount of MR plication was significantly larger than the amount of MR resection (5.1± 0.4 vs 6.2 ± 0.6, *p* < 0.001). The RR group still showed greater change of postoperative astigmatism compared to the RP group.

Because there was a significant difference in the angle of deviation and the amount of surgery depending on the type of surgery, the effects of these factors were analysed in each subgroup according to the type of surgery. In only the LRc group, the deviation angle at a distance was correlated with the changes in astigmatism after surgery. The larger the deviation angle at a distance, the greater the postoperative change toward WTR astigmatism. A larger amount of LR recession was also correlated with more changes in astigmatism, which approached statistical significance. Meanwhile, the amount of resection or plication did not correlate with the postoperative changes in astigmatism (Table 5).

**Table 5:** Mixed model using interaction terms for the factors associated with postoperative changes in astigmatism in subgroups according to the type of strabismus.

Variables	*p*-value for the mixed model		
	RR	RP	LRc
**Deviation angle**			
at distance*	0.550	0.683	**< 0.001**
at near*	0.138	0.806	0.176
**Amount of surgery**			
LR recession^†^	0.618	0.966	0.076
** MR resection/plication**	0.893	0.998	N/A

Abbreviations: RR, unilateral lateral rectus recession-medial rectus resection; RP, unilateral lateral rectus recession-medial rectus plication; LRc, unilateral lateral rectus recession.

## Discussion

The present study investigated the long-term refractive changes after unilateral horizontal muscle surgery for IXT by comparing the refractive error between both eyes. We found that the changes in SER did not differ between the operated and fellow eyes, whereas astigmatism was more changed toward WTR astigmatism in the operated eyes. The difference in astigmatism changes between both eyes was 0.25 D, which remained stable without further progression from three months after surgery.

Although many studies regarding postoperative SER have suggested an initial myopic shift after horizontal muscle surgery, it remains controversial as to whether the myopic shift is transient or permanent [7–10]. Previous studies have compared the refractive error between the preoperative and immediate postoperative periods to isolate the surgical effect on refractive error. For this reason, few studies have focused on the long-term effects of surgery on refractive error. Moreover, they had a limitation in that there was no control group for the natural course of refractive errors since they only evaluated the changes in refractive error before and after surgery. In our study, by comparing the longitudinal changes in refractive error between the operated and fellow eyes after unilateral strabismus surgery, the multifactorial effect on refractive error was eliminated, allowing us to evaluate the long-term effect of strabismus surgery on refractive error. There was no significant difference in the change in SER between both eyes, and it can be inferred that surgically induced myopia is clinically insignificant at three months after surgery.

As found in our study, an increase in WTR astigmatism after horizontal muscle surgery has also been commonly reported in several studies [6, 8, 18, 19]. Hong and Kang described astigmatic changes toward WTR after surgery in Korean children with IXT, which was sustained six months postoperatively. They reported the change of about 0.22 to 0.30 D in astigmatism, which was similar to the results of our study. Moreover, we found that this change persisted without deterioration up to four years after surgery. This implies that the surgery might have initial effects on the change in astigmatism within three months and not have additional impact thereafter.

Several previous studies have suggested that there is no significant difference in the magnitude of refractive change after various surgical procedures [8, 12, 20]. They showed no difference in the postoperative refractive changes between eyes that underwent single muscle recession and those that underwent recession-resection. Our results also showed similar astigmatism changes between the RR and LRc groups.

However, previous studies have not included eyes with plication. To the best of our knowledge, this is the first study to measure the effect of plication on postoperative refractive error, and the results showed that RP had less of an effect on astigmatism than RR or LRc. Considering that the plication procedure is added to LRc in the RP, this result is interesting. This is partially due to the difference in the amount of recession. The amount of recession was greater in the LRc group than in the RP group. Moreover, all subjects underwent recession of at least 8.5 mm in the LRc group and less than 8.5 mm in the RP group. Although many studies have reported no correlation between the magnitude of surgery and refractive change, Chun *et al*. showed a larger change after maximal recession compared with conventional recession in postoperative week one [21]. In addition, our LRc group showed that the amount of recession was correlated with the magnitude of astigmatism change with borderline significance. Therefore, a maximal recession should be considered a risk factor for changes in astigmatism.

However, in the comparison of the postoperative astigmatism change between the RR and RP groups only in the patients whose deviation angle was ≧ 20 PD and < 40 PD, the RR group still showed greater change compared to the RP group by 0.37 D (95% CI, 0.17–0.56 D), although there was no difference in the amount of the LR recession. Moreover, the amount of LR recession was not correlated with the postoperative change of astigmatism in the RR and RP groups, where 94.5% of the patients underwent LR recession by 5 – 8mm. Therefore, the conventional surgical amount would not be able to explain the difference of postoperative astigmatism between the RR and RP groups, and the distinct features of plication should also be considered a protective factor for the change in astigmatism. After recession, the extraocular muscle tension is reduced, causing corneal flattening in the quadrant of the recessed muscle [3]. During resection procedure, the extraocular muscle was disinserted and resected. Then, the muscle was reattached to the sclera using the suture that intrasclerally pass through the entire length of the muscle insertion. In contrast to resection, the extraocular muscle was not disinserted in plication procedure. Furthermore, tucking the muscle using the suture tied to the edges of the muscle insertion could compensate the corneal flattening induced by muscle recession. This may prevent postoperative astigmatism changes.

This study had several limitations. First, due to the retrospective nature of the study, we were unable to obtain data on potential confounding factors for refractive error such as family history, outdoor activities, or near work. Instead, we compared the operated and fellow eyes of each patient, thereby eliminating the effects of the factors. Second, manifest refraction was measured during the postoperative period, in contrast to preoperative cycloplegic refraction. This can result in a larger change in refractive error immediately after surgery than the actual change. This prevented us to evaluate the immediate postoperative change in refractive error. However, this limitation can be overcome by comparing both eyes undergoing the same test at each visit. We evaluated the change in refractive error induced by the strabismus surgery comparing both eyes, not comparing pre- and postoperative refractive error. Therefore, the errors induced by the non-cycloplegic refraction would have not had effects on the results of this study. Third, according to the type of surgery, the angle of deviation differed and that the amount of surgery was different, which may have influenced the result. However, there was no significant correlation between the conventional amount of surgery and the postoperative change of refractive error. Therefore, this would have little effect on the results. Forth, since none of our subjects showed an oblique axis of astigmatism, we were not able to investigate the postoperative change in oblique axis of astigmatism. Finally, we have performed RP for IXT treatment since 2013. Therefore, there was a difference in the duration of surgery between the subgroups according to the type of surgery. However, the surgeon had over 20 years of surgical experience, so the difference in the time of surgery would have little effect on the results.

In summary, horizontal muscle surgery had no long-term effect on myopia progression. Mild WTR astigmatism was induced and sustained after surgery, and RP had less of an effect on astigmatism than RR or LRc. Although the results suggest that strabismus surgery seems to have minimal long-term effects on changes in refractive error, postoperative changes in astigmatism were sustained. Therefore, clinicians should be aware of this change, and the refractive error should be checked and corrected.

## Supporting information

S1 Data (XLSX)
